# Changes in vector species composition and current vector biology and behaviour will favour malaria elimination in Santa Isabel Province, Solomon Islands

**DOI:** 10.1186/1475-2875-10-287

**Published:** 2011-09-30

**Authors:** Hugo Bugoro, Charlie Iro'ofa, Donna O Mackenzie, Allen Apairamo, Watson Hevalao, Sarah Corcoran, Albino Bobogare, Nigel W Beebe, Tanya L Russell, Cheng-Chen Chen, Robert D Cooper

**Affiliations:** 1 National Vector Borne Disease Control Programme, Ministry of Health, Honiara, Solomon Islands; 2 Institute of Tropical Medicine, National Yang-Ming University, No. 155, Sec.2, Li-Nong Street, Taipei 112, Taiwan; 3 Australian Army Malaria Institute, Gallipoli Barracks, Enoggera, 4052, Australia; 4 School of Biological Sciences, University of Queensland, Goddard Building, St.Lucia, Qld 4068, Australia; 5 The University of Queensland, School of Population Health, Australian Centre for Tropical and International Health, Pacific Malaria Initiative Support Centre, Herston, 4006, Australia

## Abstract

**Background:**

In 2009, Santa Isabel Province in the Solomon Islands embarked on a malaria elimination programme. However, very little is known in the Province about the anopheline fauna, which species are vectors, their bionomics and how they may respond to intensified intervention measures. The purpose of this study was to provide baseline data on the malaria vectors and to ascertain the possibility of successfully eliminating malaria using the existing conventional vector control measures, such as indoor residual spraying (IRS) and long-lasting insecticidal nets (LLIN).

**Methods:**

Entomological surveys were undertaken during October 2009. To determine species composition and distribution larval surveys were conducted across on the whole island. For malaria transmission studies, adult anophelines were sampled using human landing catches from two villages - one coastal and one inland.

**Results:**

Five *Anopheles *species were found on Santa Isabel: *Anopheles farauti, Anopheles hinesorum*, *Anopheles lungae, Anopheles solomonis*, and *Anopheles nataliae*. *Anopheles hinesorum *was the most widespread species. *Anopheles farauti *was abundant, but found only on the coast. *Anopheles punctulatus *and *Anopheles koliensis *were not found. *Anopheles farauti *was the only species found biting in the coastal village, it was incriminated as a vector in this study; it fed early in the night but equally so indoors and outdoors, and had a low survival rate. *Anopheles solomonis *was the main species biting humans in the inland village, it was extremely exophagic, with low survival rates, and readily fed on pigs.

**Conclusion:**

The disappearance of the two major vectors, *An. punctulatus *and *An. koliensis*, from Santa Isabel and the predominance of *An. hinesorum*, a non-vector species may facilitate malaria elimination measures. *Anopheles farauti *was identified as the main coastal vector with *An. solomonis *as a possible inland vector. The behaviour of *An. solomonis *is novel as it has not been previously found biting humans in any numbers. Both species appear to be short-lived, a characteristic that will limit their transmission potential. The early night feeding behaviour and a degree of outdoor biting seen in *An. farauti *and particularly in *An. solomonis *will require that their response to IRS and LLIN be closely monitored. In coastal villages, where large, favourable breeding sites allow for high numbers of *An. farauti *may require the addition of larval control to achieve elimination.

## Background

Recently, the international community has prioritized national and regional malaria elimination based on the strategy of shrinking the malaria map from the margins inwards [1]. The Solomon Islands is at the eastern edge of the distribution of malaria in the southwest Pacific, the disease is endemic throughout many parts of the country and up until the early 1990s was the leading public health problem [2]. The Solomon Islands has now entered the malaria pre-elimination stage together with 31 other countries [3]. To initiate this, the country's National Vector Borne Disease Control Programme (NVBDCP) has selected Temotu and Santa Isabel Provinces in which to commence malaria elimination programmes. In the remaining six provinces intensified control will be implemented.

Malaria eradication was attempted in Solomon Islands through a Malaria Eradication Programme (MEP) conducted during 1970 - 1975 [4]. On Santa Isabel, this programme was highly successful and elimination was nearly achieved with entry into the consolidation phase (cessation of DDT - IRS) planned for 1974 [4]. However similar success was not achieved in other parts of the country, most notably on the north coast of Guadalcanal [5]. The MEP was abandoned in 1975 and reduced to control measures and focal DDT indoor residual spraying (IRS) to contain outbreaks. The malaria rate rose during this period peaking in 1992 [2], in 1993 insecticide impregnated bed nets (ITN) were introduced [6] and in early 2000, DDT was replaced by lambdacyhalothrin (ICON) for IRS, also at this time ITNs were being replaced by long-lasting insecticidal nets (LLIN). Through these intervention methods Santa Isabel Province has maintained a continual attack on the vector for the last 40 years with the introduction of ITN and LLIN resulting in a continual fall in transmission rates from 44% in 1992 to 4.5% in 2008 [Source: SI NVBDCP data: unpublished]. The current malaria situation on Santa Isabel indicates a further reduction in malaria transmission. Passive case detection at the main hospital in the capital Buala recorded 81 cases over 33 months, January 2007 to September 2009. At least 11 of these cases were reported as being imported from other provinces - Guadalcanal and Malaita. The species composition was *Plasmodium falciparum *47% and *Plasmodium vivax *53%. A mass blood survey conducted on Santa Isabel in October 2009 screened 8552 of the local population. A total of 14 were found positive by microscopy (n = 1) and PCR (n = 13) giving a parasite prevalence rate of 0.16%; of these 92.9% were due to *P. vivax *and the remainder due to *P. falciparum *[7].

Several species of anophelines occur in the Solomon Islands, six members of the *Anopheles punctulatus *group: *Anopheles farauti *(formerly *Anopheles farauti 1*), *Anopheles irenicus *(formerly *Anopheles farauti 7*), *Anopheles hinesorum *(formerly *Anopheles farauti 2*), *Anopheles punctulatus, Anopheles koliensis*, and *Anopheles rennellensis *[8,9]; as well as three members of the *Anopheles lungae *complex: *Anopheles lungae, Anopheles solomonis*, and *Anopheles nataliae *[10]. Of these nine species, only *An. farauti, An. punctulatus *and *An. koliensis *are considered vectors of malaria [10]. With the latter two species the use of IRS and LLIN over the years appears to have eliminated *An. koliensis *and *An. punctulatus *is now uncommon with a patchy distribution [11,12]. The third species, *An. farauti*, did not respond well to IRS during the MEP and subsequent control programmes due to a change in behaviour to early evening, outdoor biting [12]. This species is now the primary vector in the Solomon Islands, being the most abundant and widespread species [13]. It is primarily a coastal species capable of breeding in brackish water, a trait that has facilitated its spread throughout small island groups [14]. *Anopheles farauti *is a member of the *An. farauti *complex within the *An. punctulatus *group and is morphologically indistinguishable from *An. hinesorum *and *An. irrenicus*, the other members of the complex found in the Solomon Islands. As *An. hinesorum *and *An. irenicus *are zoophilic, non-vectors species in the Solomon Islands, it is important to accurately identify these species as efforts may be wasted on controlling species of no medical importance.

On Santa Isabel, the last anopheline faunal survey was conducted between 1966 and 1971 [11] with the species composition updated in 1978 [13]. From these records *An. farauti *s.l., *An. punctulatus, An. koliensis, An. lungae*, *An. solomonis*, and *An. nataliae *were recorded. However, during the past 30 years it is possible that species composition and distribution may have been altered by continual pressure from the vector control programmes as well as biotic factors such as competition and dispersal of zoophilic species. Moreover, as these surveys predate the use of molecular techniques for species identification it is possible that other species may occur on the island. For example by using allozymes, *An. hinesorum *was identified from two locations around Buala in 1988 [15]. With the molecular based techniques currently available that will reliably identify the members of the *An. punctulatus *group the presence and distribution of these species on Santa Isabel can now be resolved; however no such techniques are as yet available for identifying the members of the *An. lungae *complex.

The strategies planned for malaria elimination in Santa Isabel Province are improved and more widely available diagnosis, greater accessibility to more effective treatment drugs, and vector control with LLIN and IRS aiming at reducing human vector contact, vector longevity, and vector density. To support the vector control measures information on the speciation, distribution, ecology, behaviour, and biology of the vector is important to ascertain what type of elimination measures are most appropriate and for which areas. The results of these surveys will also provide base line data for future monitoring and evaluation of the elimination programme. As this information is currently lacking for Santa Isabel entomological surveys were conducted in October 2009. This paper reports on the findings of these surveys

## Methods

### Study site

This study was conducted in Santa Isabel Province (8°14'21.66″S latitude and 159°33'27.08″ E longitude). The province is made up of the main Santa Isabel Island (200 km long by 25 km wide) and several closely associated smaller islands. A mountain range, up to 1500 m above sea level (asl), runs down the center of the island and there is a narrow coastal shelf (0.1 - 3 km wide and <20 m asl) that runs round the island; the majority of the population live in villages that lie along this coastal shelf. The climate is continuous hot/wet. There is no rainfall data specific to the province but weather stations in surrounding provinces indicate that rain occurs all year round and at a rate of about 2,500-3,000 mm per annum. The mean temperature for the region is 26-27°C on the coast and inland lowland regions. The total population is 26,500, most of whom live on the eastern end of the island around Buala the provincial capital. There is no road network and all movement is by boat.

### Species composition and distribution

Utilizing marine transport a larval survey was conducted covering most of the island. As the majority of the villages were located on the coast, the survey concentrated on this area; however inland villages were also surveyed. At each location, using local knowledge from the village people, all ground pools were checked around the villages. Using standard 250 ml dippers, larval samples were taken from all sites positive for anopheline larvae. A description of the site was made and its location geo-referenced on 1:50,000 scale maps. The anopheline larvae collected from each site were preserved in 70% ethanol in appropriately labeled vials.

### Selection of indicator villages for adult collections

Two indicator villages - Kolosori and Popoheo - were selected for studying the biology and behaviour of the adult anopheline fauna. Kolosori (8°07'13.21″ S, 159°31'49.96″E) is located on the coastal plain about 3.0 km inland and 20 m asl and Popoheo (8°05'34.18″ S, 159°31'24.10″E) on the same plain but directly on the coast (<5 m asl) and surrounded by brackish water swamps. Both villages were chosen in the vicinity of Buala for reliable access. Three houses in each of these villages were randomly selected for the indoor human landing catches. In Kolosori a host preference study was carried out comparing the attractiveness of paired human and pig baits.

### Human landing catches (HLC)

Local villagers from Buala were hired and trained to perform the human landing catches. For each indicator village, 12 collectors were hired, with six working from 6 pm to midnight and the other six working from midnight to 6 am. Collections were made for 10 nights in each indicator village.

Three collectors worked indoors and three outdoors. Each collector collected for 40 min each hour; during this period the collectors, using a torch and aspirator, caught all anophelines coming to feed on their exposed legs and feet. Specimens collected were held in separate cups labelled for each hour, either outdoors or indoors. The collectors were alternated with regards to indoor/outdoor and collection time either the first or second parts of the night. The cups were covered with damp cotton wool and held until the following morning when the mosquitoes were killed using chloroform, morphologically identified [10] and the hourly collection rates recorded. All adult mosquitoes collected were preserved, desiccated on silica gel, in microfuge tubes each labelled with the village, date, time of feeding, method of collection and whether collected indoor or outdoor.

### Longevity of vector populations

Parity was determined based on the condition of the tracheolar skeins of the ovaries [16] this was done on all anophelines collected each hour from the night landing collections. From this the proportion parous (P) was used to determined the survival through one day (*p*) as ^*x*^√P where *x *is the length of the gonotrophic cycle. The proportion of the vector population living long enough to transmit malaria parasites was determined by *p*^*n *^where n was 9 days for *P. vivax *and 12 days for *P. falciparum *[17].

### Human host behavior

A census of the people outdoors each hour from 6 pm - 6 am was conducted in each of the indicator villages on each night that the human landing catches were performed. Based on the total number people in the village, the number of people indoors each hour was then determined and related to the biting times of the anophelines.

### Host seeking in relation to host indoor/outdoor movements

The use of indoor and outdoor collectors creates an artificial situation where for example mosquitoes collected outdoors, late in the night when no village people are outdoors, are counted as outdoor biting. It is more realistic when estimating the degree of indoor (or outdoor) biting to account for the spatial interaction between the vector and the host, resulting from the movement of vectors and hosts in and out of houses. This is particularly so where the vector control tools (IRS and LLIN) being implemented only target indoor biting mosquitoes. The degree of indoor or outdoor biting will be a factor of where the people are at the time of vector host seeking. Thus the proportion of indoor biting mosquitoes (π_i_) was calculated by multiplying the number of *Anopheles *biting indoors with the number of people indoors for each hour of the night and similarly the outdoor biting component (π_o_) was multiplied by the number of people outdoors for each hour of the night. The mathematical formulae for estimating π_i _have been detailed previously [18].

### Molecular identification of mosquitoes

All specimens collected were initially identified using morphological keys [10]. As many of the *Anopheles *species in the Solomon Islands belong to isomorphic species complexes the morphological identification of the specimens collected was verified using polymerase chain reaction - restriction fragment length polymorphisms (PCR-RFLP) of the ITS2 region of the ribosomal DNA [19]. Up to 10 individual larvae from each larval collection site, and all of the adults from the human landing catches and the animal baited traps were processed. DNA extraction, amplification, restriction digest, fragment separation, and visualization are as previously described [20]. With any anomalies or unknown RFLP then the ITS2 was sequenced for that specimen and compare to the existing sequences in the GenBank [20].

### Vector incrimination

The head and the thorax of all adult mosquito specimens collected biting humans and from bait net traps were processed using specific monoclonal antibodies and an enzyme-linked immunosorbent assay (ELISA). The methods used were those supplied with the monoclonal reagents (Dr Robert Wirtz, Centers for Disease Control and Prevention, MS F42, Atlanta, GA 30341-3717, USA). Specimens were considered positive if the absorbance value was twice that of the average negative control value and all positives were rerun for confirmation.

### Animal host preference

Two animal baited traps were set up, one containing a human, the other two small pigs. To protect the human bait the person slept inside a normal untreated bed net covered by the animal bait-net. With both the human and pig traps, the skirt of the nets were raised 10 cm off the ground at 6 pm each evening to allow entry of blood seeking mosquitoes during the night and lowered at 6 am each morning. The traps were inspected at 7 am each morning and all trapped mosquitoes were collected, counted and scored for unfed or blood fed.

### Ethics

Ethical approval for the study was obtained from the University of Queensland Medical Research Ethics Committee (2010000412).

## Results

### Anopheles species identification

**Figure 1:**
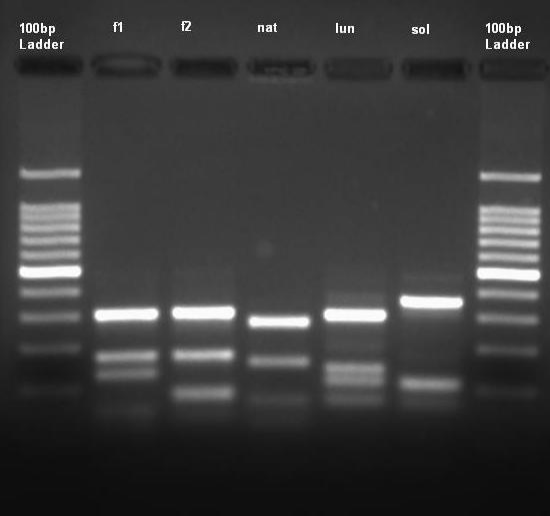
**Internal transcribed spacer 2 amplification products from: *Anopheles farauti *(f1), *Anopheles hinesorum *(f2), *Anopheles nataliae *(nat), *Anopheles lungae *(lun), and *Anopheles solomonis *(sol), digested with *Msp *I and run on a 3% agarose gel with a 100 base pair ladder**.

All adults were initially identified using morphological keys, with specimens of *An. farauti *s.l. and those of the *An. lungae *complex being identified. Following this, the application of PCR-RFLP techniques revealed the presence of *An. farauti *and *An. hinesorum*. Similar PCR techniques when applied to the specimens morphologically identified as members of the *An. lungae *complex demonstrated the presence of three species which, when sequenced and compared to material in GenBank, were found to be *An. lungae *and *An. solomonis*. For the third species there was no match but this species is most likely *An. nataliae*, the third member of this complex, as morphological and sequence similarities placed it in the *An. lungae *complex. Therefore the digest enzyme MspI used in this PCR method was able to separate not only the members of the *An. farauti *complex in the Solomon Islands but also the members of the *An. lungae *complex (Figure 1). From the larval collections 1070 specimens were processed using PCR with six species identified: *An. farauti, An. hinesorum*, *An. lungae*, *An. solomonis*, *An. nataliae*, and *Bironella hollandi*. The genus *Bironella *belongs to the subfamily Anophelinae, the larvae are macroscopically similar to *Anopheles*; this species was identified here by comparing ITS2 sequences in the Gen-Bank.

### Anopheles species distribution and larval ecology

**Figure 2:**
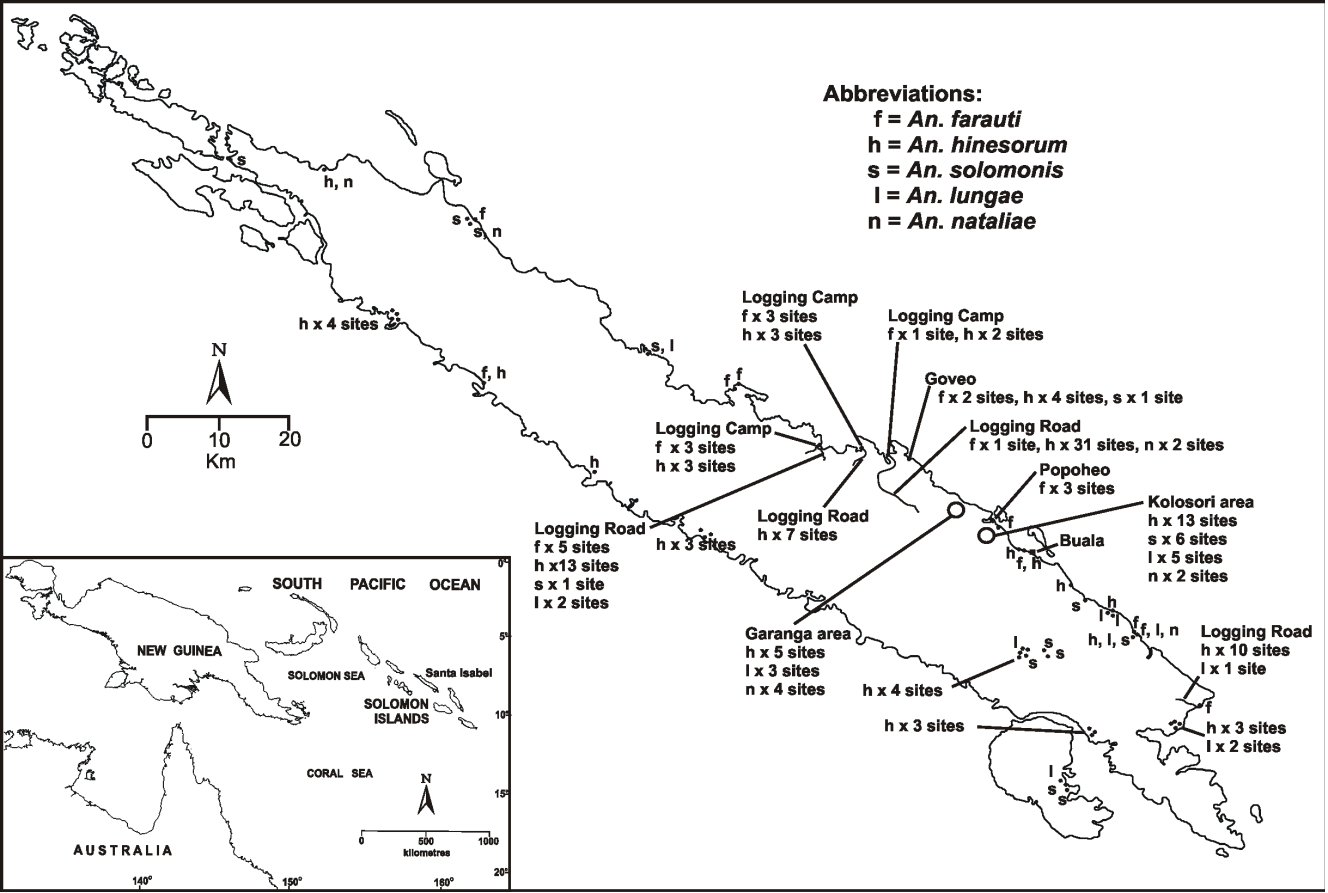
**Speciation and distribution of anopheline fauna on Santa Isabel based on larval surveys conducted in October 2009**. Insert is the map of the Southwest Pacific.

**Figure 3:**
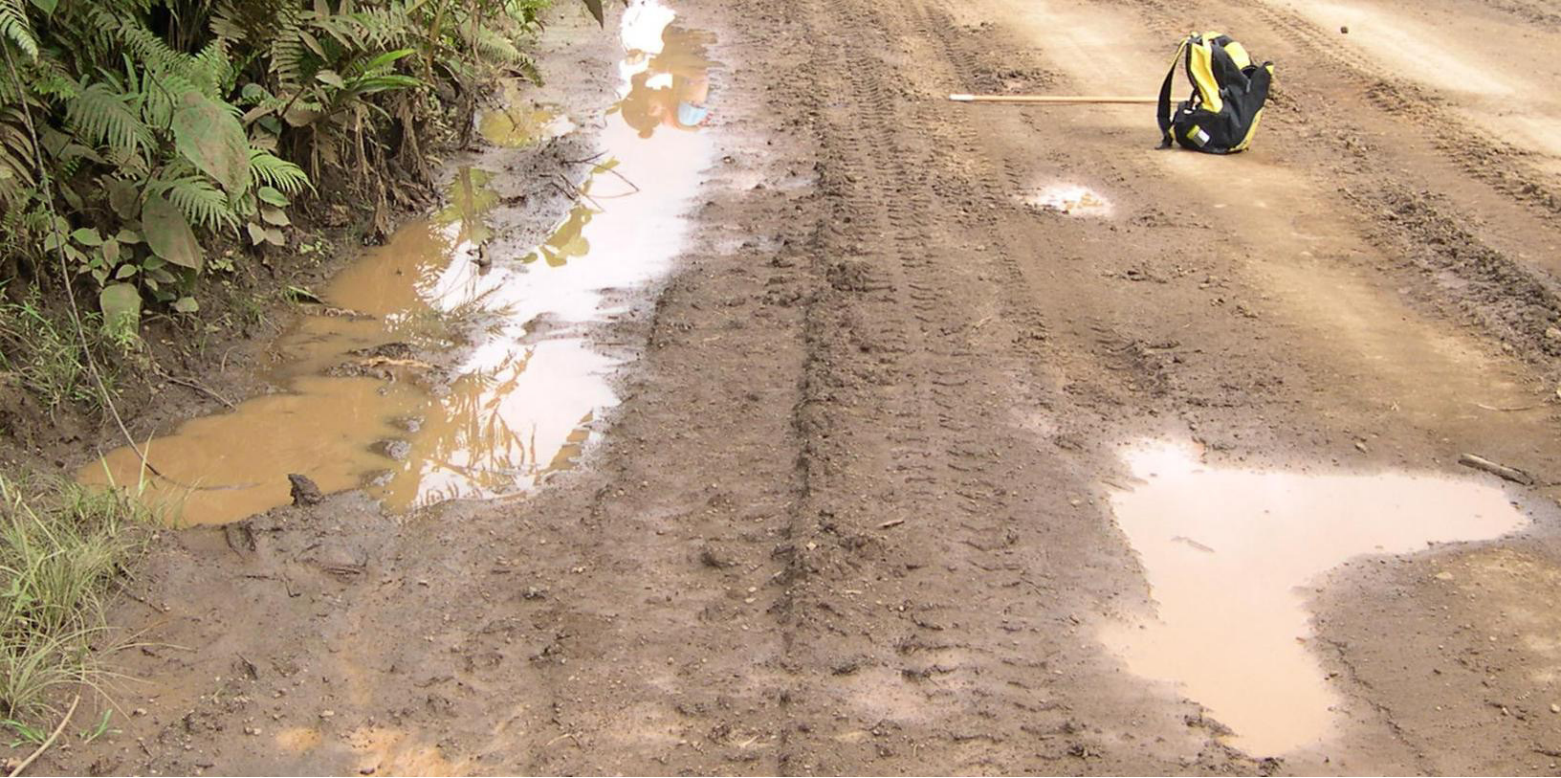
**Typical transient ground pools, in this case wheel ruts - small, turbid, with clay substrate, and devoid of any flora or fauna**. In the past these sites were used exclusively by *Anopheles punctulatus*, on Santa Isabel they are now being used exclusively by *Anopheles hinesorum*.

Anopheline larvae were collected from 146 sites throughout the island (Figure 2). *Anopheles hinesorum *was the most abundant and widespread of all species being found in 82.2% (120/146) of positive sites both inland and along the coast. *Anopheles farauti *was less common with collections from 19.9% of sites positive but with a distribution restricted to the coast. Members of the *An. lungae *complex were widespread throughout the island being found both coastal and inland but like *An. farauti *were less common than *An. hinesorum*. Breeding sites were categorized broadly into six main types (Table 1). *Anopheles hinesorum *was collected from all types of sites except tidal swamps, but was most commonly found in transient (Figure 3) and semi-permanent ground pools. *Anopheles farauti *was found in brackish water but also in fresh water habitats, all coastal. Members of the *An. lungae *complex preferred shaded, heavily vegetated sites mostly associated with drains, creeks and water courses.

**Table 1:** Aquatic larval habitats utilized by the six species found on Santa Isabel.

Type of site	Species and number of sites occupied (%)					
	
	*An. farauti*	*An. hinesorum*	*An. lungae*	*An. solomonis*	*An. nataliae*	*B. hollandi*
Swamps tidal	6 (20.7%)	0	0	0	0	0
Swamps fresh	2 (6.9%)	4 (3.3%)	3 (13.6%)	3 (15.8%)	1 (12.5%)	0
Riparian	0	6 (5.0%)	8 (36.4%)	7 (36.8%)	3 (37.5%)	5 (83.3%)
Drains	6 (20.7%)	27 (22.5%)	7 (31.8%)	9 (47.4%)	1 (12.5%)	0
Ground pools, semi-permanent	11 (37.9%)	40 (33.3%)	3 (13.6%)	0	3 (37.5%)	1 (16.7%)
Ground pools, transient	4 (13.8%)	43 (35.8%)	1 (4.5%)	0	0	0

Total	29	120	22	19	8	6

Where riparian sites included pools in creek lines and the margins along creeks usually heavily vegetated and shaded. Drains were all earthen, slow flowing, usually heavily vegetated and shaded. Semi-permanent ground pools refers to small to medium pools of water well established with vegetation in and around the site, well developed aquatic flora and fauna, usually shaded. Examples: borrow pits, large vehicle tracks, blocked creeks and water courses, natural rain filled depressions. Transient ground pools were those maintained only by regular rainfall they were unestablished with no vegetation or fauna usually exposed, unshaded and with a clay substrate. Examples: vehicle tracks, small natural depressions, pig wallows, foot prints, ruts in roads and foot tracks (Figure 3).

### Species biting humans and their biting density in two indicator villages

In Popoheo, the only species collected during HLC was *An. farauti*. Over the 10 nights of collection, 775 *An. farauti *were collected, with a landing rate of 12.9/person/night (Table 2).

**Table 2:** Human landing collections of *An. farauti *and host movements in Popoheo village.

Time pm-am	No. collected outdoors	No. collected indoors	% of total catch	% of villagers outdoors
6-7	100	101	25.9	68.7
7-8	104	100	26.3	51.1
8-9	54	43	12.5	35.4
9-10	39	38	9.9	24.0
10-11	27	25	6.7	13.4
11-12	21	22	5.5	7.6
12-1	2	4	0.8	10.0
1-2	3	5	1.0	10.1
2-3	9	2	1.4	8.1
3-4	5	3	1.0	11.0
4-5	7	4	1.4	13.8
5-6	32	25	7.4	26.4

Totals	403	372	100	

Collections were made each hour (6 pm-6 am) over 10 nights by three collectors indoors and three collectors outdoors and the percentage of the village population outdoors for each hour (based on a mean of 10 nights and a total village population of 70).

At the inland village of Kolosori, 304 anophelines were collected in ten nights of human landing catches. These were identified by PCR as 285 *An. solomonis*, 10 *An. farauti*, and nine *An. hinesorum*. Thus, the most common anopheline biting humans in this inland village was *An. solomonis *with a landing rate of 4.75/person/night (Table 3).

**Table 3:** Human landing collections of *An. solomonis *and host movements in Kolosori village.

Time pm-am	No. collected outdoors	No. collected indoors	% of total catch	% of villagers outdoors
6-7	130	17	51.6	100
7-8	26	15	14.4	85.7
8-9	21	4	8.8	66.0
9-10	13	2	5.3	36.5
10-11	10	2	4.2	28.2
11-12	9	3	4.2	22.7
12-1	5	0	1.8	19.5
1-2	7	2	3.2	13.2
2-3	1	1	0.7	14.8
3-4	2	2	1.4	12.7
4-5	1	0	0.4	11.8
5-6	12	0	4.2	22.5

Totals	237	48	100	

Collections were made each hour (6 pm-6 am) over 10 nights by three collectors indoors and three collectors outdoors and the percentage of the village population outdoors for each hour (based on a mean of 10 nights and a total village population of 60).

### Biting behaviour

In Popoheo, the majority of *An. farauti *host seeking occurred early in the evening (Table 2) with 52.3% of the mosquito population active between 6 - 8 pm. Biting virtually ceased after mid-night except for a pre dawn peak between 5 - 6 am. Equal numbers of *An. farauti *fed indoors and outdoors even early in the evening between 6 - 9 pm when 68.7 - 35.4% of the village population was outdoors. Over the ten nights the average outdoor to indoor biting ratio was 1:0.93.

In Kolosori, the majority of *An. solomonis *host-seeking took place early in the evening (Table 3) with 51.2% of the mosquitoes collected in the first hour of the night (6 - 7 pm) and 74.2% of biting occurring within the first three hours of the night. This peak biting period of *An. solomonis *coincided with the highest number of people outdoors (Table 3). This early night feeding and a predominantly outdoor human population at this time of the evening (Table 3) allows for a high degree of outdoor biting and the overall outdoor to indoor biting ratio for the ten nights was 1:0.2.

### Interaction between human hosts and vectors

In Popoheo, the biting times of *An. farauti *were correlated with the movement of people indoors during the night to provide a more realistic assessment of the amount of indoor biting (π_i_). This indicated that on average, each night, 54.6% of biting occurred indoors. For *An. solomonis *in Kolosori, adjusting for the movement of people outdoors to indoor during the night indicated that only 6.9% of biting occurred indoors.

### Longevity of the mosquito populations

Age grading, by estimating the proportion of the mosquito population that had previously laid at least one batch of eggs, was carried out at Popoheo for *An. farauti*. The overall proportion parous for this species was 0.411 (n = 632 dissected) as the duration of the gonotrophic cycle is 2.5 days for *An. farauti *in the Solomon Islands [21] the daily survival rate was 0.70. The extrinsic phase (time for the malaria parasite to develop in the mosquito) for *P. vivax *and *P. falciparum *is 9 and 12 days respectively at 26-27°C [17], the mean coastal temperature for most parts of the Solomon Islands [22]. Thus with this population of *An. farauti *only 4.0% and 1.4% of mosquitoes would live long enough to transmit *P. vivax *and *P. falciparum *respectively.

In Kolosori, the overall proportion parous for *An. solomonis *was 0.333 (n = 221 dissected) assuming a similar duration for the gonotrophic cycle this equates to a daily survival rate of 0.64. Thus with this population of *An. solomonis *only 1.8% and 0.47% of mosquitoes would live long enough to transmit *P. vivax *and *P. falciparum *respectively..

### Host preference

In Kolosori, a direct comparison was made between the number of *Anopheles *species coming to bite humans and pigs. Over 10 nights, 44 *An. solomonis *and two *An. hinesorum *were collected off pigs but none of either species were collected off the paired human bait. This indicated that while this species will feed on humans, as was shown in the HLC results, it also has a preference for non-human hosts.

### Detection of circumsporozoite antigen and EIR

In Popoheo, 775 specimens of *An. farauti *were assayed for circumsporozoite antigen with one positive for *P. vivax *(210 variant). The specimen was collected indoors between 9-10 pm. The sporozoite rate for *An. farauti *in Popoheo village was 0.00129, with a biting rate of 12.9 bites/person/night this represents an EIR of 6.07 infective bites/person/year (Table 4).

**Table 4:** The entomological estimation of malaria transmission intensity attributable to *Anopheles farauti *on Popoheo village and *Anopheles solomonis *on Kolosori village, Santa Isabel province, Solomon Islands during October of 2009.

Entomological parameters of mosquito population	Popoheo *Anopheles farauti*	Kolosori *Anopheles solomonis*
Sporozoite prevalence (S; %)	0.00129 (*n *= 775)	0.0000 (*n *= 287)

Biting rate (B; b/p/n)		
Indoor	12.4	1.6
Outdoor	13.4	8.0
Overall	12.9	5.1

Entomological inoculation rate ib/p/y	6.07	<0.001

Endophagy^1 ^Proportion indoors ± se	0.46 ± 0.04 (*n *= 775)	0.21 ± 0.06 (*n *= 287)

Nocturnal biting^2 ^Proportion 10 pm-5 am ± se	0.03 ± 0.01 (*n *= 775)	0.02 ± 0.01 (*n *= 287)

Proportion indoor biting ^3 ^(π_i_)	0.546	0.069

Where S = no. of sporozoite positive mosquitoes/no. of mosquitoes tested, B = no. of mosquitoes collected/no. of nights/no. of collectors, EIR = S × B_overall _× 365. Endophagy was the proportion of mosquitoes caught indoors (calculated as *I*_6 pm_→_5 am_/(*I*_6 pm_→_5 am _+ *O*_6 pm_→_5 am_, where *I *and *O *= the total number of mosquitoes caught indoors and outdoor respectively between 6 pm and 5 am); nocturnal biting was the proportion of mosquitoes caught during hours (10 pm-5 am) when most people are asleep (calculated as *I*_9 pm_→_4 am _+ *O*_9 pm_→_4 am_)/(*I*_6 pm_→_5 am _+ *O*_6 pm_→_5 am _where *I *and *O *= the total number of mosquitoes caught indoors and outdoor respectively at the time indicated); and π_i _the proportion of the vector population biting indoors adjusted for the location of the host either outdoors or indoors [12]

In Kolosori, 304 specimens were tested for circumsporozoite antigens. Of the 304 specimens, 285 of them were *An. solomonis*, 10 *An. farauti*, 9 *An. hinesorum *and all were negative.

### Malaria prevalence in the two indicator villages

Of the 14 positive cases detected during the mass blood survey of November 2009, two *P. vivax *cases occurred in Popoheo (0.91% [2/219]) and one *P. vivax *case in an adjacent coastal village Hovikoila 1.5 km to the north. No malaria cases were found in Kolosori village. In May 2010 four cases of *P. vivax *were detected by PCD in Popoheo village [7]. These findings indicate a persistent but low rate of malaria transmission is occurring in the study area.

## Discussion

A major finding of this work is the absence of *An. punctulatus *and *An. koliensis *from Santa Isabel. These two primary vectors of malaria were recorded on the island in 1970, at the time *An. punctulatus *was uncommon, but *An. koliensis *was common and widespread [8]. Their disappearance is most likely due to years of IRS, first with the MEP and then the on-going control programme. This occurred in New Guinea and in other parts of the Solomon Islands when IRS was introduced, though with the cessation of the eradication programmes or a reduction in IRS coverage they rebounded [11,12,23,24].

A further factor which may have helped with their elimination is competition from *An. hinesorum*; which was found to be dominant anopheline species on Santa Isabel. *Anopheles hinesorum *occupied breeding sites commonly used by *An. koliensis*, particularly drains and semi-permanent ground pools. It was also commonly found in transient ground pools (Figure 3). These sites are normally used exclusively by *An. punctulatus *[14] to the point where when *An. farauti *and *An. koliensis *larvae were seeded into these types of sites they failed to develop [25]. *Anopheles hinesorum *populations in the Solomon Islands are zoophilic and are not involved in malaria transmission [26,27]. The disappearance of *An. punctulatus *and *An. koliensis *and their replacement by a zoophilic, non-vector species is likely responsible for some of the decline in transmission rates, particularly in inland villages.

*Anopheles farauti*, the third major malaria vector in the Solomon Islands, was common on Santa Isabel, it was found predominantly on the coast where the majority of the human population is located. The larvae of *An. farauti *will develop in brackish water and as found here in Popoheo and recently in coastal villages in Temotu Province large brackish coastal swamps are the most productive sites for this species; these site can be responsible for high human biting densities [21].

In this study, *An. farauti *was incriminated as a vector for vivax malaria in Popoheo, and is most likely the main vector on Santa Isabel. There have been problems in the past with the control of this species. Of concern is its shift to early night outdoor feeding thought to be due to an excito-repellent response to the IRS. This change in biting behaviour allowed it to avoid the insecticide and maintain transmission outdoors limiting the effectiveness of past eradication and control programmes [5,12,28]. In Popoheo *An. farauti *was found to be an early night feeder with 52.3% of host seeking occurring between 6 - 8 pm, however it was still quite endophagic and readily enter houses to feed even during the early hours of the night when considerable numbers of villagers were outdoors. This is anomalous with *An. farauti *populations in other parts of the Solomon Islands where similar intervention measures resulted in outdoor/indoor feeding ratios of 1:0.25 [12,15]. Whether on Santa Isabel the withdrawal of IRS, with only occasional focal spraying, has removed the insecticide pressure allowing for a return to indoor biting is not known. Adjusting outdoor and indoor biting for the movement of people from outdoors to indoors during the night, it is estimated that 54.6% of the biting of *An. farauti *occurs indoors each night and that this proportion of the vector population could be controlled if good coverage of IRS and LLIN was achieved in the implementation of the elimination programme.

In the inland village of Kolosori, *An. solomonis *was recorded as the dominant human biting anopheline. This is the first time this species has been found biting humans in appreciable numbers, prior to this there was very little evidence for considering this species as a possible malaria vector [10]. *Anopheles solomonis *had a pronounced early night feeding pattern and it was extremely exophagic. This type of behavior would make this species difficult to control with IRS or LLIN. The results of the host preference study indicated that *An. solomonis *would readily feed on pigs (4.6/night) with no specimens collected off the paired human bait, though at the same time the night biting catches recorded an overall biting rate of 4.75 b/p/n. A mass blood survey conducted in Kolosori village at the same time as the entomological survey found no positive malaria cases and all (285) human biting *An. solomonis *assayed for circumsporozoite antigen were negative.

Kolosori was the only place in the Solomon Islands to date where *An. hinesorum *has been found biting humans; this was in very low numbers (2.96% [9/304]), particularly in light of the number of larval sites positive for this species (13 sites) in the immediate vicinity of Kolosori village. This species is inconsequential with regards to malaria transmission. These finding support previous work on this species in the Solomon Islands where it has been identified as a zoophilic species [26,27].

The malaria vector *An. farauti *was also found biting humans in inland Kolosori but in small numbers (3.29% [10/304]). The flight range for this species is up to 3 kilometers [29], and in the absence of *An. farauti *larvae in the Kolosori area it is most likely that these few specimens have come in off the coast. From a malaria transmission point of view, these low numbers are unlikely to contribute to malaria transmission, though the possible continued presence of this species in these inland villages should be monitored. The proportion parous was low for both *An. farauti *and *An. solomonis*, being 0.41, and 0.33 respectively. These values are indicative of short-lived populations with only a very few specimens living long enough to transmit the parasite. For *An. farauti *in other parts of its range in the Solomon Islands and Papua New Guinea considerably higher survival rates, ranging from 0.55 to 0.80, have been recorded [4,30].

Unlike the provinces of Guadalcanal and Malaita, where malaria is more intense, the disease does not seem to be well entrenched on Santa Isabel. Since the MEP of the early 1970s, continued use of focal IRS, and the introduction of insecticide treated nets in 1993, has seen a decline in the malaria cases with an overall parasite rate of 0.26% being recorded in 2009. The mass blood survey conducted in October 2009 estimated a similarly low overall parasite rate of 0.16% [7]. The reason for the decline in malaria rates is most likely to be due to the elimination of *An. koliensis *and *An. punctulatus *from Santa Isabel leaving *An. farauti *as the only recognized vector. This species can be an efficient vector of malaria and in villages on Guadalcanal it has been responsible for maintaining parasite rates of 30-46% [30]. However on Santa Isabel, despite reasonable biting densities at Popoheo, the sporozoite rate was 0.00129 and only two human cases were found in the village during the mass blood survey of 2009 [7]. It is possible that there is sufficient indoor host-seeking to result in some degree of *An. farauti *exposure to insecticide treated nets or IRS which may be responsible for the low survival rate of this species on Santa Isabel and thus its poor vectoring ability. It was found on Guadalcanal, that the introduction of insecticide-treated nets resulted in a parous rate in *An. farauti *of 0.39 as compared to 0.55 in a no-net site and 0.57 in a DDT IRS site [30].

With *An. farauti *restricted to the coast and the disappearance of *An. koliensis *and *An. punctulatus*, the inland villages (>3 kilometers from the coast) of Santa Isabel should be malaria free unless *An. solomonis *plays some role in transmission. Even if this species was capable of transmitting the parasites, its low biting densities, a degree of zoophilia, and very low survival rates would make it a particularly inefficient vector.

In the Solomon Islands, factors that have been shown to play a lead role in the decline of malaria rates are IRS, LLIN, and education [2]. On Santa Isabel all three of these factors are currently implemented. Additionally the communities on Santa Isabel have a national reputation for a high level of active participation and support for self-betterment. Such community attitudes promote high levels of acceptance for activities like malaria awareness and education plus support and good co-operation for the implementation of IRS and LLIN intervention measures.

On Santa Isabel, malaria elimination is being sought through LLIN distribution to all households in all villages throughout the province in addition to complete and thorough IRS coverage in all houses in all villages along the coast targeting the distribution of the main vector *An. farauti*. This is a realistic approach as IRS is very labour intensive and costly and just focusing on the main vector allows for a concentration of resources where most needed. Additional to vector control there will be better access to diagnosis and treatment with the recent introduction of a new treatment drug Coartem^® ^(artemether + lumefantrine) to replace chloroquine which has become increasingly ineffectual due to *P. falciparum *and *P. vivax *resistance. The effect of these measures on the already declining parasite rate should see the achievement of malaria elimination from Santa Isabel though the impact of the IRS and LLIN on *An. farauti *will have to be monitored as will the possible role *An. solomonis *might play in transmission in inland villages.

## Conclusion

With regards to the vectors, there are a number of factors that will favour the success of renewed efforts in malaria elimination on Santa Isabel. The disappearance of the competent malaria vectors *An. punctulatus *and *An. koliensis *and interspecific competition against all species from *An. hinesorum*, a zoophilic, non-vector species. The restriction of the only recognized vector, *An. farauti*, to the coast will allow for elimination strategies to be focused and intensified in the coastal areas. The indoor biting activity (despite its early night feeding behaviour) of *An. farauti *will enhance the effectiveness of IRS while the low survival rate of this species will be a mitigating factor against high rates of transmission. The finding of *An. solomonis *as a possible inland vector should be monitored, but its low survival rates and zoophilic tendencies may limit its ability to be an effective vector. The impact of IRS and/or LLIN on both *An. farauti *and *An. solomonis *will require close monitoring as both species show varying degrees of early night outdoor biting, particularly in *An. solomonis*, also *An. farauti *has been found, in other part of the Solomon Islands, to shift to early outdoor biting under insecticidal pressure.

## Competing interests

The authors declare that they have no competing interests.

## Authors' contributions

Conceived and designed the experiments: HB, RDC, AB. Performed the experiments: HB, RDC, TLR, SC, DOM, CI, AA, WH. Performed the molecular analysis: NB, DOM. Analyzed the data and wrote the manuscript: HB, RDC, TLR. Reviewed the manuscript: AB, DOM, TLR, CCC. All authors have read and approved the final manuscript.
